# Proteinase 3 promotes formation of multinucleated giant cells and granuloma-like structures in patients with granulomatosis with polyangiitis

**DOI:** 10.1136/ard-2021-221800

**Published:** 2023-02-17

**Authors:** Scott R Henderson, Harry Horsley, Paul Frankel, Maryam Khosravi, Talya Goble, Stephen Carter, Marilina Antonelou, Rhys D R Evans, Xiang Zhang, Tai-Ying Chu, Hsi-Hsien Lin, Siamon Gordon, Alan David Salama

**Affiliations:** 1 UCL Department of Renal Medicine, Royal Free Hospital, London, UK; 2 UCL Institute of Cardiovascular Science Faculty of Population Health Sciences, London, UK; 3 Department of Cell and Developmental Biology, UCL Division of Biosciences, London, UK; 4 Department of Microbiology and Immunology, Chang Gung University, Taoyuan, Taiwan; 5 Department of Anatomic Pathology, Chang Gung Memorial Hospital at Linkou, Taoyuan, Taiwan; 6 Graduate Institute of Biomedical Sciences, Chang Gung University College of Medicine, Taoyuan, Taiwan; 7 Sir William Dunn School of Pathology, Oxford University, Oxford, UK

**Keywords:** granulomatosis with polyangiitis, systemic vasculitis, autoimmune diseases

## Abstract

**Objectives:**

Granulomatosis with polyangiitis (GPA) and microscopic polyangiitis (MPA) are autoimmune vasculitides associated with antineutrophil cytoplasm antibodies that target proteinase 3 (PR3) or myeloperoxidase (MPO) found within neutrophils and monocytes. Granulomas are exclusively found in GPA and form around multinucleated giant cells (MGCs), at sites of microabscesses, containing apoptotic and necrotic neutrophils. Since patients with GPA have augmented neutrophil PR3 expression, and PR3-expressing apoptotic cells frustrate macrophage phagocytosis and cellular clearance, we investigated the role of PR3 in stimulating giant cell and granuloma formation.

**Methods:**

We stimulated purified monocytes and whole peripheral blood mononuclear cells (PBMCs) from patients with GPA, patients with MPA or healthy controls with PR3 or MPO and visualised MGC and granuloma-like structure formation using light, confocal and electron microscopy, as well as measuring the cell cytokine production. We investigated the expression of PR3 binding partners on monocytes and tested the impact of their inhibition. Finally, we injected zebrafish with PR3 and characterised granuloma formation in a novel animal model.

**Results:**

In vitro, PR3 promoted monocyte-derived MGC formation using cells from patients with GPA but not from patients with MPA, and this was dependent on soluble interleukin 6 (IL-6), as well as monocyte MAC-1 and protease-activated receptor-2, found to be overexpressed in the cells of patients with GPA. PBMCs stimulated by PR3 formed granuloma-like structures with central MGC surrounded by T cells. This effect of PR3 was confirmed in vivo using zebrafish and was inhibited by niclosamide, a IL-6-STAT3 pathway inhibitor.

**Conclusions:**

These data provide a mechanistic basis for granuloma formation in GPA and a rationale for novel therapeutic approaches.

**WHAT IS ALREADY KNOWN ON THIS TOPIC**

- Although the structure of the granulomas and multinucleated giant cells in granulomatosis with polyangiitis (GPA) is known, little is established about what drove granuloma formation and why it is infrequently found in patients with myeloperoxidase-antineutrophil cytoplasm antibodies.
- Clinical association of augmented interleukin 6 (IL-6) levels in patients with GPA has been reported.

**WHAT THIS STUDY ADDS**

- The study demonstrates a key role for persistent proteinase 3 (PR3) stimulation of monocytes promoting giant cell and granuloma formation and a difference in the PR3 binding partners between patients with GPA and those with microscopic polyangiitis.
- The study also demonstrates that the process is IL-6-dependent, which potentially explains the clinical links with augmented IL-6 found in patients with GPA.
- In addition, it provides a novel animal model of PR3-mediated granuloma formation, which has been lacking to date.

**HOW THIS STUDY MIGHT AFFECT RESEARCH, PRACTICE OR POLICY**

- The study has provided a number of potential therapeutic strategies that may work better in GPA and a novel animal model that could allow for screening of novel compounds and a better molecular understanding of the PR3-mediated process.

## Introduction

Multinucleated giant cells (MGCs) form during chronic inflammation as a result of persistent antigenic stimulation due to various infectious organisms (such as *Mycobacterium tuberculosis*), chronic particle exposure (such as silicosis) and in various autoimmune inflammatory diseases, some with and some without known antigens.^1^ Antineutrophil cytoplasm antibody (ANCA)-associated vasculitis (AAV) is an autoimmune small vessel inflammatory disease associated with ANCA and specific immune reactivity against neutrophil and monocyte proteinase 3 (PR3) or myeloperoxidase (MPO).^2^ AAV results in two main clinical syndromes: granulomatosis with polyangiitis (GPA) and microscopic polyangiitis (MPA). Patients with GPA with ANCA generally demonstrate reactivity to PR3, with less frequent MPO reactivity in European populations.^3 4^ GPA is characterised by granulomatous inflammation most frequently found in the upper and lower airway and in the eye.^5 6^ Lesions frequently recur and are notoriously difficult to treat, requiring prolonged immunotherapy.^7 8^ Genome-wide association studies have demonstrated genetic differences underlying MPO-associated and PR3-ANCA-associated disease, with the former being linked with HLA-DQ alleles and the latter with HLA-DP, and genes encoding PR3 (*PNTR3*) and alpha-1 antitrypsin (A1AT) (*SERPINA1*).^9 10^

Granulomas contain fibroblasts interspersed with leucocytes, specifically T cells, and monocytes, with MGCs located at the core.^11^ The stimuli for monocyte and macrophage fusion are diverse and may be dependent on the persistence of particular antigens, as well as cytokine-driven macrophage activation^12 13^ and expression of particular macrophage molecules.^12^ In GPA, less is known about MGC formation or the reason for the close association with PR3-ANCA. However, neutrophil infiltration followed by generation of apoptotic cells or necrotic debris and microabscesses precedes MGC lining sites of inflammation.^14^ Unlike, MPO-ANCA-associated MPA, in which rodent models have been very successfully developed,^15–18^ rodent models of PR3-ANCA disease, and specifically the granulomatous manifestations, have universally failed to replicate human disease.^19 20^ This is thought to be in part due to differences between human and rodent PR3 biology.^21^

PR3 is a serine protease, structurally related to human neutrophil elastase (HNE), cathepsin G and enzymatically inactive azurocidin, and is highly expressed on neutrophils and at lower levels on monocytes and macrophages.^22^ PR3 demonstrates a genetically regulated bimodal distribution on the surface of resting neutrophils, unlike HNE or MPO.^23–25^ While both PR3 and MPO act as autoantigens in systemic vasculitis, HNE-ANCA has only been implicated in a proportion of patients with cocaine-induced vasculitis, and then often in association with PR3-ANCA, and is not found in idiopathic GPA or MPA.^26^ Following neutrophil apoptosis, PR3, unlike HNE, continues to be highly expressed, functioning as an inflammatory molecule rather than an innate microbicidal protein,^27^ frustrating macrophage phagocytosis and altering macrophage polarisation,^27^ independently of its enzymatic activity.^27 28^ PR3 can bind and activate monocyte cell surface receptors, particularly protease-activated receptor-2 (PAR-2) through enzymatic cleavage, mediating interleukin (IL)-6, IL-8 and IL-1 cytokine release,^29^ as well as interacting with macrophage-1 antigen (MAC-1) and calreticulin.^30 31^

Since patients with GPA have augmented cellular PR3 expression and predominantly display autoreactivity towards PR3, we tested the hypothesis that in GPA it is persistent PR3 that promotes MGC formation. We generated an in vitro model to investigate the cellular mechanisms driving monocyte fusion following PR3 exposure and tested this in a novel in vivo *Danio rerio* model.

## Methods

### Study participants

Patients with GPA and MPA were identified from our clinical vasculitis database, with the disease classified according to the Chapel Hill Consensus Conference diagnostic criteria.^5^ Patient demographics, clinical characteristics and investigations including ANCA reactivity were documented from electronic records. Disease activity, scored by the Birmingham Vasculitis Activity Score (BVAS) and the Vasculitis Damage Index (VDI), was calculated.^32 33^

### PBMC and monocyte isolation

Peripheral blood mononuclear cells (PBMCs) were isolated from whole blood and purified monocytes were obtained by CD14-positive selection using magnetic bead isolation (Miltenyi Biotec, UK). Phenotyping of monocytes was performed by flow cytometry (see online supplemental methods). CD14-positive cells were stimulated in complete culture medium and 10% heat-inactivated human AB serum (Sigma, UK), in the presence of antigens and relevant inhibitors (see online supplemental methods), all run in duplicate. Experiments for generating MGC followed previously reported methods.^34 35^

10.1136/ard-2021-221800.supp1 Supplementary video

### Calculation of monocyte fusion index

Samples were stained with a modified Giemsa stain. Light microscopy was performed and images were analysed using ImageJ software (see online supplemental methods) to calculate the proportion of cells forming MGC.

### Cytokine analysis

Cytokine cytometric bead assays (LEGENDplex, BioLegend, UK) were performed to measure the levels of individual cytokines in culture supernatants, according to the manufacturer’s instructions. Culture supernatant IL-6 concentration was tested by ELISA (R&D, UK) in other experiments.

### In vivo *D. rerio* model

The transgenic Tg(mpeg:GFP) line was kindly provided by Professor Maggie Dallman (Imperial College London, UK). Briefly, zebrafish embryos were injected into the yolk sac with PR3 or human albumin, intact or heat-inactivated, at either 1 pg/mL and 5 pg/mL, and the embryos were imaged 5 days later (see online supplemental methods).

### Statistics

Statistics were performed using GraphPad Prism V.8 software. Categorical variables were tested using χ^2^ tests. Median values with 95% CIs or IQR were reported. Rates of monocyte fusion were tested by Mann-Whitney U tests for two variables or by one-way or two-way analysis of variance (ANOVA) for three or more. Significance was defined by a p value <0.05.

### Patient and public involvement

Patients and the public were not formally consulted regarding this study.

## Results

### Subjects

A total of 34 patients with GPA (30 with PR3-ANCA and 4 with MPO-ANCA), 10 patients with MPA (all with MPO-ANCA) and 10 healthy controls (HCs) were recruited. Demographics were recorded for all patients (online supplemental results table 1). The BVAS and the VDI specifically showed more ocular, ENT (ear, nose and throat) and pulmonary manifestations in patients with GPA compared with renal involvement in patients with MPA.

**Table 1:** Effect of PR3 and albumin injection at different doses in zebrafish with or without niclosamide administration to the fish tank water

Antigen/dose	1 pg/mL	P value	5 pg/mL	P value
Albumin	22 997 (20 454–32 491)			
PR3	28 479 (22 970–48 552)	<0.05 albumin vs PR3		
Hi albumin	22 005 (19 918–24 273)		22 713 (19 918–31 486)	ns intact vs Hi albumin
Hi PR3	31 163 (23 793–46 733)	<0.001 Hi albumin vs Hi PR3	31 571 (23 137–49 647)	<0.001 albumin vs PR3; ns intact vs Hi PR3
Inhibition experiments				
PR3 alone	106 969 (41 031–465 184)			
PR3+niclosamide 0.01	117 473 (35 128–245 781)	ns vs PR3 alone		
PR3+niclosamide 0.05	63 043 (27 395–205 060)	<0.05 vs PR3 alone		
PR3+niclosamide 0.15	44 501 (26 861–90 354)	<0.001 vs PR3 alone		
PR3+niclosamide 0.3	42 730 (25 638–324 957)	<0.05 vs PR3 alone		

Hi, heat-inactivated; PR3, proteinase 3.

### MGC formation in patients with GPA is promoted by PR3

Isolated monocytes were consistently >90% pure, based on CD14 staining (data not shown). To confirm fusion rather than cell coalescence, cultured cells were washed prior to fixing in methanol and stained with Giemsa. MGC formation was first assessed with concanavalin A-conditioned media, which confirmed typical morphological appearances of well-defined cellular aggregates (see online supplemental figure 1).

**Figure 1:**
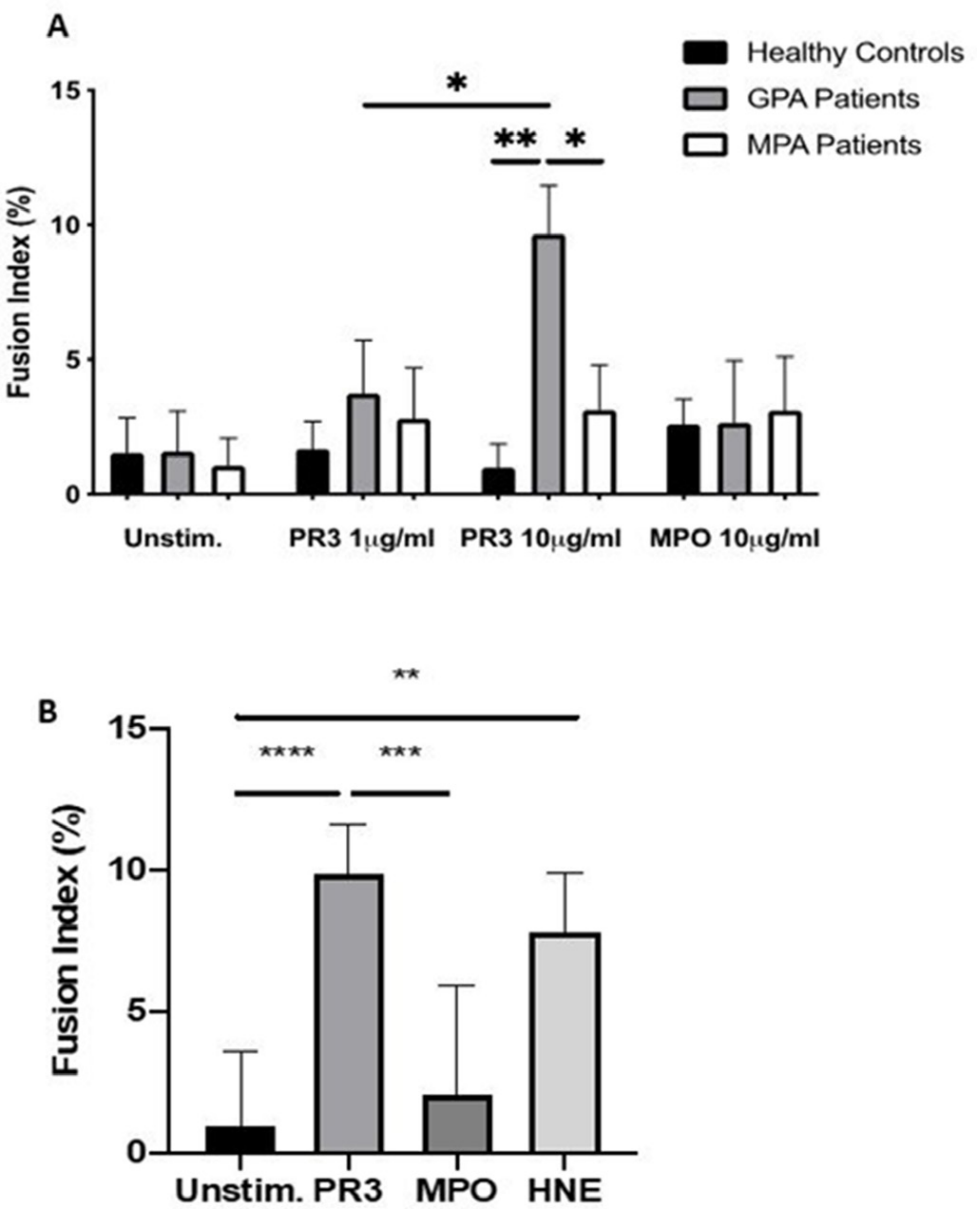
PR3 induces MGC formation in monocytes from patients with GPA. Monocytes from patients with GPA, patients with MPA and healthy controls were stimulated with PR3 and MPO (n=10) (A). There was a dose–response effect seen in patients with GPA with increasing MGC formation at PR3 10 µg/mL compared with patients with MPA and healthy controls. MPO did not stimulate MGC formation. HNE was also tested (B) and showed a similar increase in MGC formation in patients with GPA. Values plotted as median and 95% CI; *p<0.05, **p<0.01, ***p<0.001, ****p<0.0001. Difference between conditions determined by one-way and two-way ANOVA tests. ANOVA, analysis of variance; GPA, granulomatosis with polyangiitis; HNE, human neutrophil elastase; MGC, multinucleated giant cells; MPA, microscopic polyangiitis; MPO, myeloperoxidase; PR3, proteinase 3; unstim, unstimulated.

PR3 (1 µg/mL and 10 µg/mL) and MPO (10 µg/mL) were then tested on monocytes isolated from patients with GPA, patients with MPA and HC (figure 1A). There was a dose-dependent MGC formation in patients with GPA with PR3 (median fusion index: 1 µg/mL 3.17 (CI 2.35 to 5.68) and 10 µg/mL 9.86 (CI 7.01 to 11.6)), which was absent in patients with MPA (2.23 (CI 1.22 to 4.8) and 2.8 (CI 1.73 to 4.79)) or in HC (1.37 (CI 0.62 to 2.88) and 1.17 (CI 0.14 to 1.33)) for 1 µg/mL and 10 µg/mL, respectively (n=10 in all groups; p<0.05, two-way ANOVA). There was minimal MGC formation when cells were unstimulated and no significant MGC formation in any group stimulated with MPO. In addition, HNE was tested on the monocytes of patients with GPA (figure 1B) and it also promoted MGC formation (9.3 (CI 5.71 to 11.11); n=6, p<0.01, two-way ANOVA test vs unstimulated cells).

**Figure 2:**
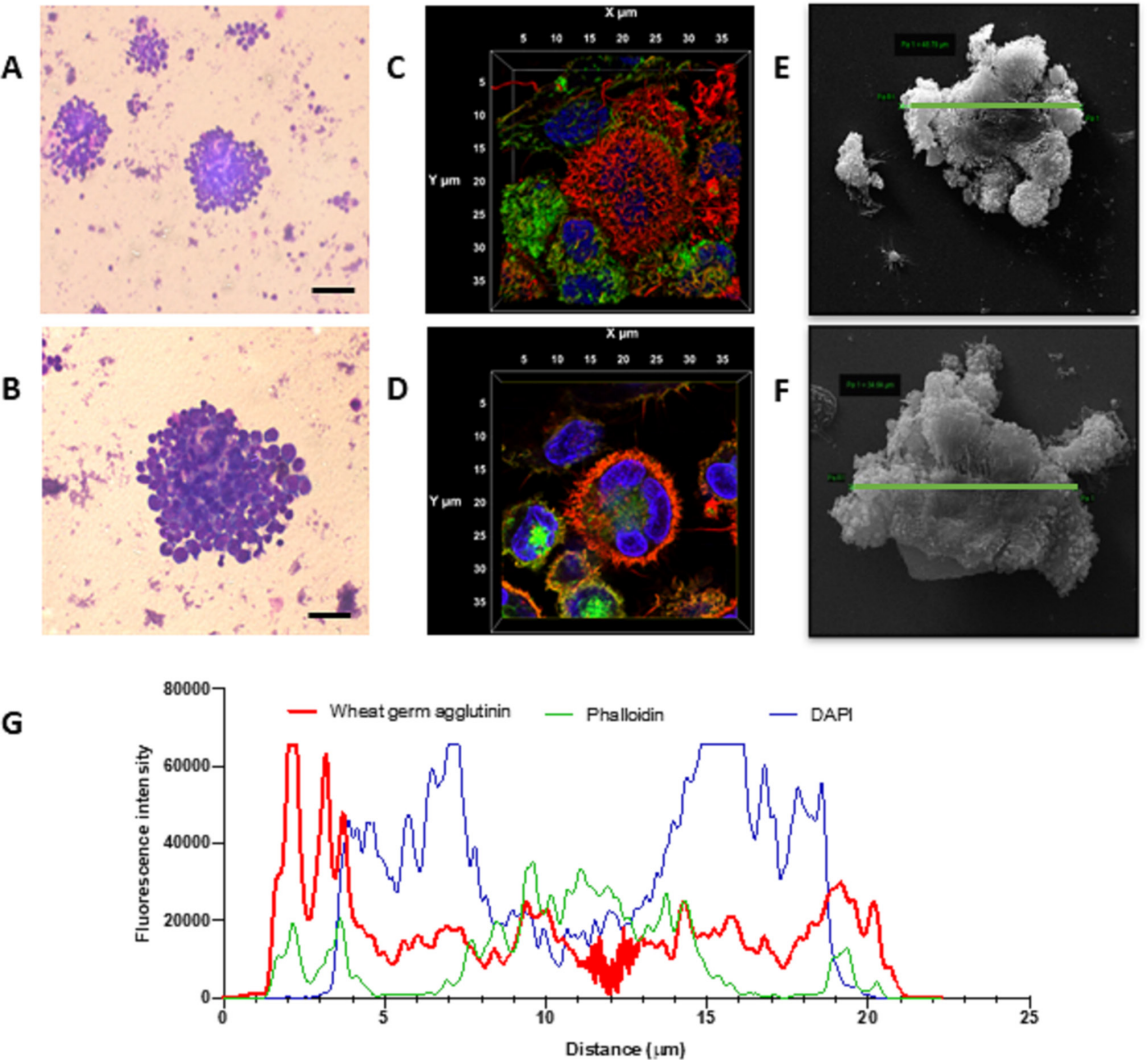
Monocyte fusion is stimulated by PR3. Monocytes stimulated with PR3 were first imaged by Giemsa staining. Distinct cellular aggregates formed in the presence of PR3 (A). At higher magnification (B), a larger cellular aggregate is present showing dense nuclear staining and cytoplasmic fusion (scale bars 100 µm). Three-dimensional volume rendering of an aggregate, including all acquired slices by confocal microscopy, was undertaken using cell membrane (red), cytoskeletal (green) and nuclear staining (blue) (C, D). Midslice (D) confirmed membrane fusion, cytoskeletal rearrangement and a single cellular structure with fusion of three monocytes, defining an MGC. Scanning electron microscopy highlighted monocyte fusion, with representative images showing an MGC size of 48.8 µm (E) and 34.6 µm (F). Membrane fusion was measured by profile plot of the fluorescence intensity of the three channels over the x/y diameter (G). There was prominent membrane staining encasing cytoskeleton and three nuclei. MGC, multinucleated giant cells; PR3, proteinase 3.

MGC formation was further confirmed by confocal microscopy using combined staining of cell membrane (using wheat germ agglutinin), actin (using phalloidin) and nuclear staining (using 4′,6-diamidino-2-phenylindole (DAPI)) (n=3), as well as by scanning electron microscopy (figure 2A–F). Giemsa staining demonstrated classic morphological features of MGC, with a large cellular aggregate showing dense nuclear staining and cytoplasmic fusion (figure 2A, B). Confocal microscopy with three-dimensional rendering demonstrated prominent outer membrane staining (figure 2C), with fused cells contained within a single cellular structure (figure 2D). Orientation of membrane and cytoskeletal and nuclear staining was also measured by profile plot of three channels of fluorescence intensity over the x/y diameter (figure 2G). Finally, MGC diameters were measured by scanning electron microscopy and ranged in size from 34.6 μm to 48.8 µm (mean 37 µm) (figure 2E, F).

### PR3-induced MGC formation is associated with IL-6 and MCP-1 production

**Figure 3:**
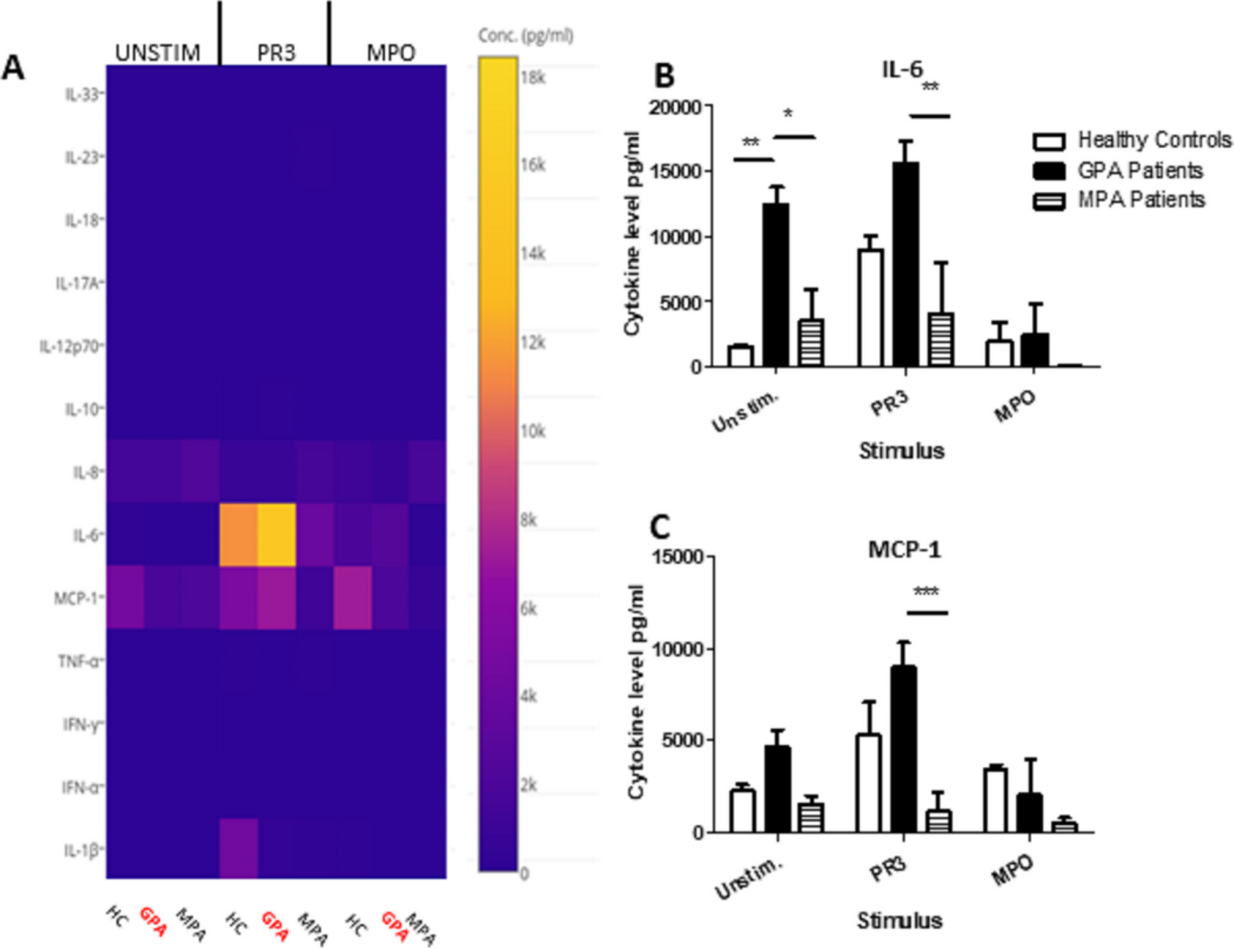
PR3-induced MGC stimulates proinflammatory cytokines. Cytokines and chemokines were measured in MGC cell culture supernatants in unstimulated, PR3-stimulated or MPO-stimulated cells (A). IL-6 production increased in the presence of PR3 in patients with GPA compared with patients with MPA, but was not significantly different from HC (B). A reduction in IL-6 production was seen in patients with GPA following stimulation with MPO. Monocyte Chemoattractant protein (MCP)-1 production (C) was also increased in patients with GPA following stimulation with PR3 compared with patients with MPA. MPO stimulation did not have any effect on differences in IL-6 or MCP-1 production. Values plotted as median and IQR; *p<0.05, **p<0.01, ***p<0.001. Difference between multiple conditions determined by two-way ANOVA. ANOVA, analysis of variance; GPA, granulomatosis with polyangiitis; HC, healthy controls; IL, interleukin; IFN, Interferon; MGC, multinucleated giant cells; MPA, microscopic polyangiitis; MPO, myeloperoxidase; PR3, proteinase 3; TNF, tumour necrosis factor.

Cytokine arrays were used to quantify the cytokines and chemokines in unstimulated, PR3-stimulated or MPO-stimulated MGC culture supernatants using monocytes from HC, patients with GPA or patients with MPA (n=4 in all groups) (figure 3A). IL-6 was produced most in unstimulated monocytes from patients with GPA (unstimulated median IL-6: GPA 12 379 pg/mL (IQR 9999–14 992), HC 1419 pg/mL (IQR 1231–1806), MPA 2020 pg/mL (IQR 23–8637); GPA vs HC p<0.01, GPA vs MPA p<0.05, two-way ANOVA) (figure 3B). Following PR3 stimulation, monocytes from patients with GPA augmented IL-6 significantly more than patients with MPA, but this was not significantly different from HC (median IL-6: GPA 15 952 pg/mL (IQR 12 281–18 398), HC 8956 (IQR 6916–10 946), MPA 114 (IQR 31–11 850); GPA vs MPA p<0.01, two-way ANOVA) (figure 3B). Following MPO stimulation, there was no statistical change in IL-6 production. For monocyte chemoattractant protein (MCP)-1, unstimulated GPA cells produced the most MCP-1, but this was not statistically different from the cells from HCs or patients with MPA (median MCP-1: GPA 9161 pg/mL (6379–11 422), HC 4362 (2486–8928), MPA 48 (37–3252)). However, following PR3 stimulation, GPA cells incremented MCP-1 production the most (median MCP-1: GPA 4488 pg/mL (IQR 3124–6445), HC 2045 (IQR 1809–2960), MPA 1593 (IQR 663–2398); GPA vs MPA p<0.001, GPA vs HC ns, two-way ANOVA) (figure 3C). No significant changes were seen following MPO stimulation among the three groups. In addition, no significant differences were found in the levels of tumour necrosis factor-alpha (TNF-α), Interferon (IFN)-α, IFN-γ, IL-10, IL-8, IL-17, IL-12, IL-18, IL-23 or IL-33 with or without stimulation.

### Modulation of MGC formation in the cells of patients with GPA

**Figure 4:**
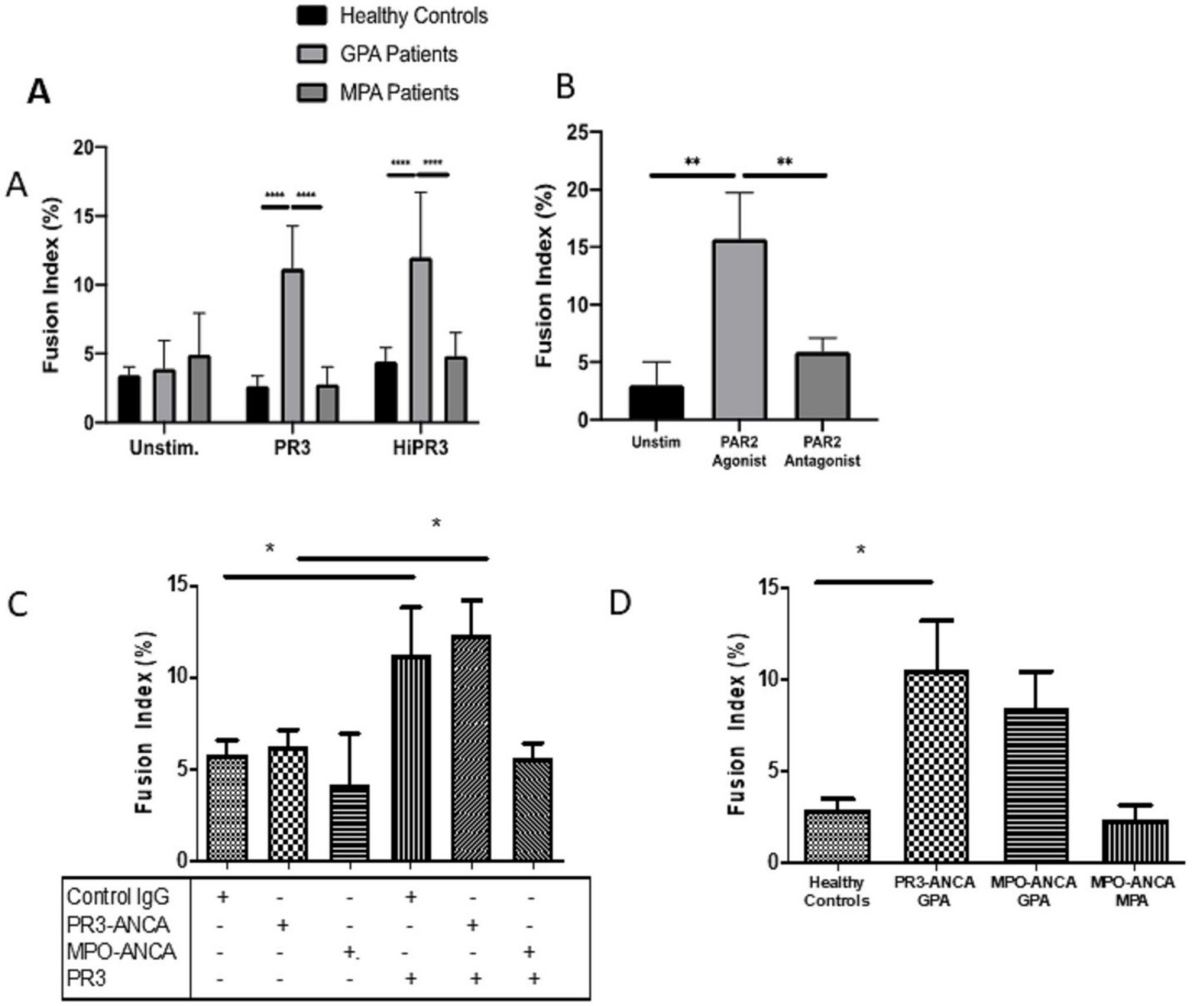
MGC forms in the presence of enzymatically active and inactive PR3. No statistical difference in fusion index was seen in the cells from patients with GPA when stimulated with enzymatically active or heat-inactivated (Hi) PR3 (A). PAR-2 agonism increased the fusion index in patients with GPA, and PAR-2 antagonism significantly inhibited the rates of MGC formation (B). The presence of PR3-ANCA or MPO-ANCA alone had no effect on fusion index compared with control immunoglobulin (C); however, when cultured with PR3, there was a significant increase in MGC formation in the presence of PR3-ANCA and control immunoglobulin compared with MPO-ANCA. Patients with GPA with different ANCA subtypes (D). Patients with PR3-ANCA GPA showed a significant increase in monocyte fusion compared with healthy controls and patients with MPO-ANCA MPA; however, a similar increase in MGC formation was seen in patients with MPO-ANCA GPA. Values plotted as median and 95% CI; *p<0.05, **p<0.01, ***p<0.001, ****p<0.0001. Difference between conditions determined by one-way and two-way ANOVA tests. ANCA, antineutrophil cytoplasm antibodies; ANOVA, analysis of variance; GPA, granulomatosis with polyangiitis; MGC, multinucleated giant cells; MPA, microscopic polyangiitis; MPO, myeloperoxidase; PAR-2, protease-activated receptor-2; PR3, proteinase 3; unstim, unstimulated.

PR3 enzymatic activity can mediate binding and cleavage of PAR-2, activating monocytes, which can be inhibited by A1AT. In addition, non-enzymatic antigenic activity may be mediated through binding receptors such as MAC-1 or calreticulin. Isolated monocytes from patients with GPA (n=8) were cultured with enzymatically active or inactive PR3 (online supplemental figure 2) and fusion index calculated as before. Both enzymatically active and heat-inactivated PR3 produced significant MGC formation in the cells of patients with GPA compared with unstimulated cells (p<0.01, two-way ANOVA) (figure 4A). We then tested the effect of a PAR-2 agonist (SLIGRL-NH2; 200 µM) and antagonist (FSLLRY-NH2; 200 µM) in combination with PR3 on MGC formation. PR3 and PAR-2 agonists increased fusion compared with unstimulated conditions (p<0.001, one-way ANOVA), and in the presence of the antagonist PR3-mediated MGC formation was inhibited down to unstimulated levels (p<0.001, one-way ANOVA) (figure 4B).

Since human AB serum used in the culture media contained A1AT (measured at 1.2 g/L by the Department of Biochemistry, Royal Free Hospital), we repeated experiments using serum-free medium (X-VIVO) and PR3 stimulation. Spontaneous and PR3-mediated rates of MGC formation were not significantly different using serum-free media. Moreover, addition of exogenous human A1AT (2 g/L) alone or in the presence of PR3 did not reduce MGC formation (online supplemental figure 3).

The effect of PR3-ANCA or MPO-ANCA on MGC formation was also tested (figure 4C). There was no effect of isolated PR3-ANCA or MPO-ANCA on MGC formation compared with control immunoglobulin; however, in the presence of PR3, PR3-ANCA and control immunoglobulin both increased the rates of MGC formation compared with MPO-ANCA (p<0.01, one-way ANOVA).

### Patients with GPA with MPO-ANCA subtype

Less commonly, a granulomatous (GPA) phenotype is seen in patients who express MPO-ANCA rather than PR3-ANCA. These patients present clinically like patients with PR3-ANCA GPA, although they are more often female, with less severe disease manifestations.^36–38^ We therefore compared MGC formation using cells from patients with GPA with MPO-ANCA positivity (n=4). Again, patients with PR3-ANCA GPA showed greater MGC fusion compared with HC and patients with MPO-ANCA MPA when monocytes were cultured with PR3 10 µg/mL (p<0.05, one-way ANOVA) (figure 4D). Patients with MPO-ANCA GPA showed a similar preponderance to MGC formation as patients with PR3-ANCA GPA, suggesting that ANCA reactivity was less critical in mediating granulomatous phenotype.

### MAC-1 and PAR-2 expressions are increased on monocytes from patients with GPA and along with IL-6 are critical for MGC formation

**Figure 5:**
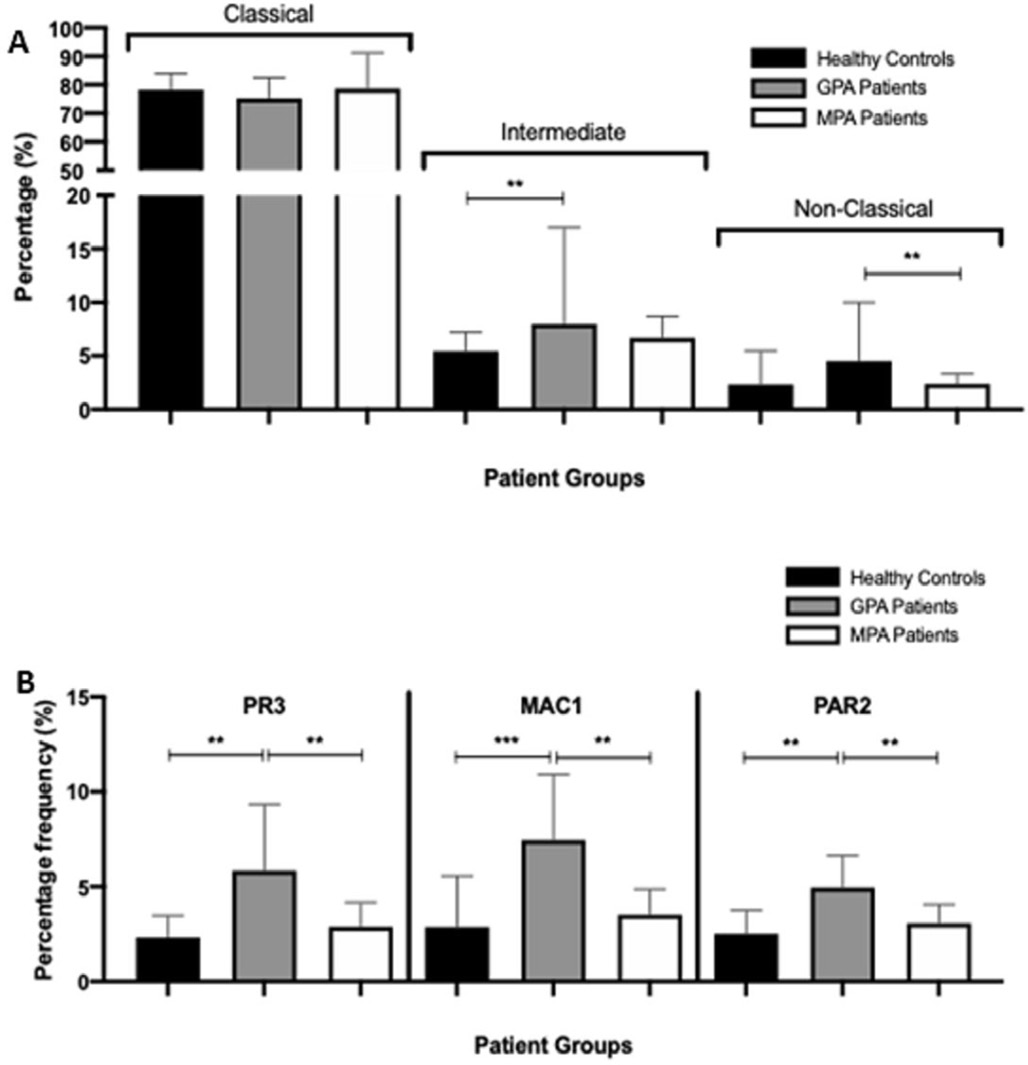
PR3, MAC-1 and PAR-2 expression is increased on the monocytes from patients with GPA. Following staining, the proportions of classic (CD14hi CD16lo), intermediate (CD14hi CD16hi) and non-classic (CD14lo CD16hi) monocytes were calculated. There was a significant increase in intermediate monocytes in patients with GPA compared with healthy controls and non-classic monocytes in patients with GPA compared with patients with MPA (A). PR3, MAC-1 and PAR-2 expression on the monocytes of patients with CD14-positive GPA was significantly increased compared with healthy controls and patients with MPA (B). Values plotted as median and 95% CI; *p<0.05, **p<0.01, ***p<0.001. Difference between conditions determined by one-way and two-way ANOVA tests. ANOVA, analysis of variance; GPA, granulomatosis with polyangiitis; MAC-1, macrophage -1 antigen; MPA, microscopic polyangiitis; PAR-2, protease-activated receptor-2; PR3, proteinase 3.

Since PR3 promotes MGC formation more readily in patients with GPA rather than in patients with MPA, we investigated the differences in monocyte populations and cell surface binding partners between patients with GPA and those with MPA. As we found both enzymatically active and inactive PR3 may promote MGC formation, and since PR3 may bind cell surface MAC-1,^30^ we tested for differences in monocyte phenotype and cell surface expression of MAC-1, PAR-2 receptor and gp130 (IL-6R beta) (online supplemental figure 3) in GPA, MPA and HC (n=6). There was no difference in frequency of the classic monocyte subsets between groups but an increase in intermediate monocytes in patients with GPA compared with HC (p<0.01, one-way ANOVA) and non-classic monocytes in patients with GPA compared with patients with MPA (p<0.01, one-way ANOVA) (figure 5A). The percentage of monocyte cell surface PR3 expression was increased in patients with GPA (median 5.78%, IQR 4.74–6.68) compared with HC (3.47%, IQR 3.15–3.47) and patients with MPA (3.44%, IQR 3.31–4.13) (both p<0.01, one-way ANOVA) (figure 5B). We found no difference in the levels of gp130 (IL-6R beta) expression on CD14+ cells between patients with MPA and those with GPA (data not shown). Monocyte MAC-1 expression was increased in patients with GPA (6.76%, IQR 6.27–7.74) compared with HC (3.55%, IQR 2.89–3.95) (p<0.001) and patients with MPA (3.73%, IQR 3.3–3.87) (p<0.01, one-way ANOVA). Similarly, PAR-2 cell surface expression on monocytes was significantly increased in patients with GPA (4.79%, IQR 4.64–5.09) compared with HC (2.98%, IQR 2.05–3.71) and patients with MPA (3.21%, IQR 2.92–3.77) (both p<0.01, one-way ANOVA). To demonstrate that MAC-1-expressing and PAR-2-expressing cells were the ones that formed giant cells, we isolated by flow cytometry CD14+PAR-2+MAC-1+ cells and compared them with CD14+PAR-2−MAC-1− cells and showed that the former induced a significantly greater degree of giant cell formation (online supplemental figure 7). We went on to confirm that PR3 is capable of binding MAC-1 using transfected HEK cells (online supplemental figure 8).

**Figure 6:**
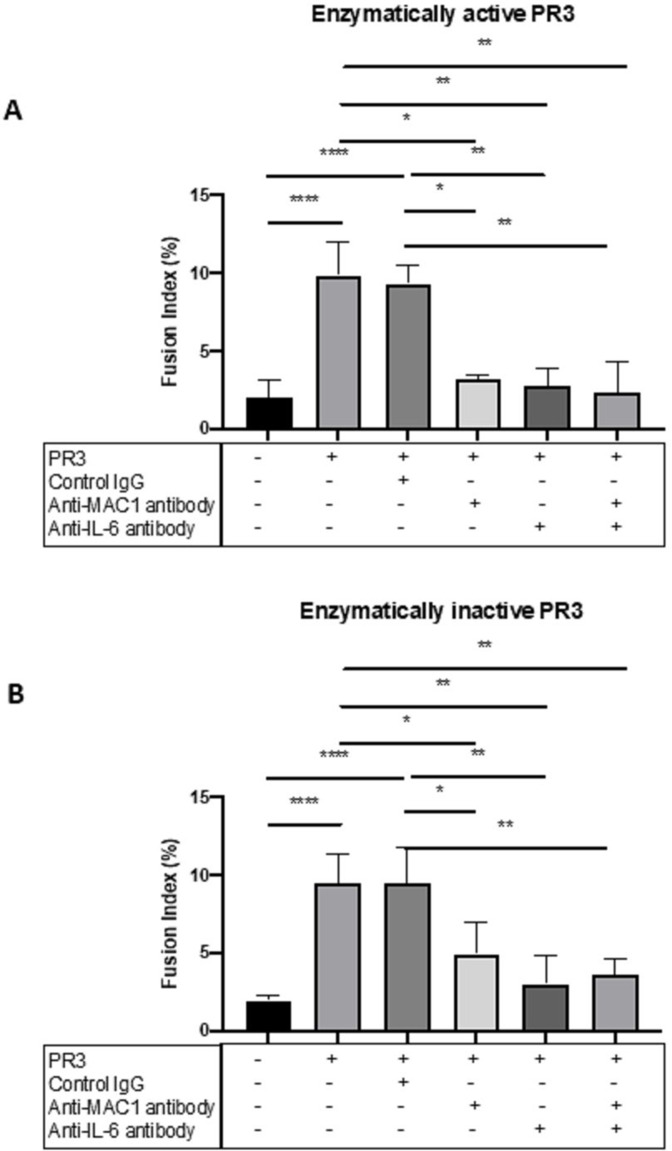
MGC formation is dependent on MAC-1, PAR-2 and IL-6. The effect of MAC-1 and IL-6 inhibition was tested in patients with GPA by culturing monocytes with enzymatically active or inactive PR3 and measuring MGC formation in the presence or absence of anti-MAC-1 antibody, anti-IL-6 antibody or control immunoglobulin. Consistently, PR3 stimulated an increase in MGC formation as well as in the presence of control immunoglobulin with enzymatically active and inactive PR3 (A, B). Anti-MAC-1 antibody and anti-IL-6 antibody reduced MGC formation in both conditions, and the effect of combined anti-MAC-1 antibody and anti-IL-6 antibody also significantly reduced MGC formation. Values plotted as median and 95% CI; *p<0.05, **p<0.01, ***p<0.001, ****p<0.0001. Difference between conditions determined by one-way ANOVA tests. ANOVA, analysis of variance; IL, interleukin; MGC, multinucleated giant cells; PAR-2, protease-activated receptor-2; PR3, proteinase 3.

As the importance of blocking the enzymatic effect of PAR-2 was already identified, we tested the effect of anti-MAC-1 and anti-IL-6 antibodies in the presence of enzymatically active or inactive PR3 in patients with GPA (n=4) (figure 6A, B). Enzymatically active and inactive PR3 were again associated with a significant increase in MGC formation using cells from patients with GPA compared with unstimulated cells (p<0.001, one-way ANOVA) and the presence of control immunoglobulin had no effect. However, anti-MAC-1 antibody significantly reduced MGC formation in enzymatically active and inactive PR3-stimulated monocytes (p<0.05, one-way ANOVA), as did anti-IL-6 antibody (p<0.01, one-way ANOVA). The combined effect of anti-MAC-1 and anti-IL-6 antibodies was a similar reduction in MGC formation, suggesting that both PR3 binding both PAR-2 and MAC-1 can mediate MGC formation and is IL-6-dependent.

### Granuloma-like structures form following PR3 stimulation of PBMC

Whole PBMCs isolated from three different patients with GPA were stimulated with PR3 (10 µg/mL) and cultured for different time points up to 3 days (online supplemental figures 5 and 6). By 3 days, extensive cell fusion was visible and confocal imaging demonstrated an inner core of CD14-positive cells surrounded by an outer core of CD3-positive T cells, mimicking a formed granuloma.

### In vivo effect of PR3 on MGC formation

**Figure 7:**
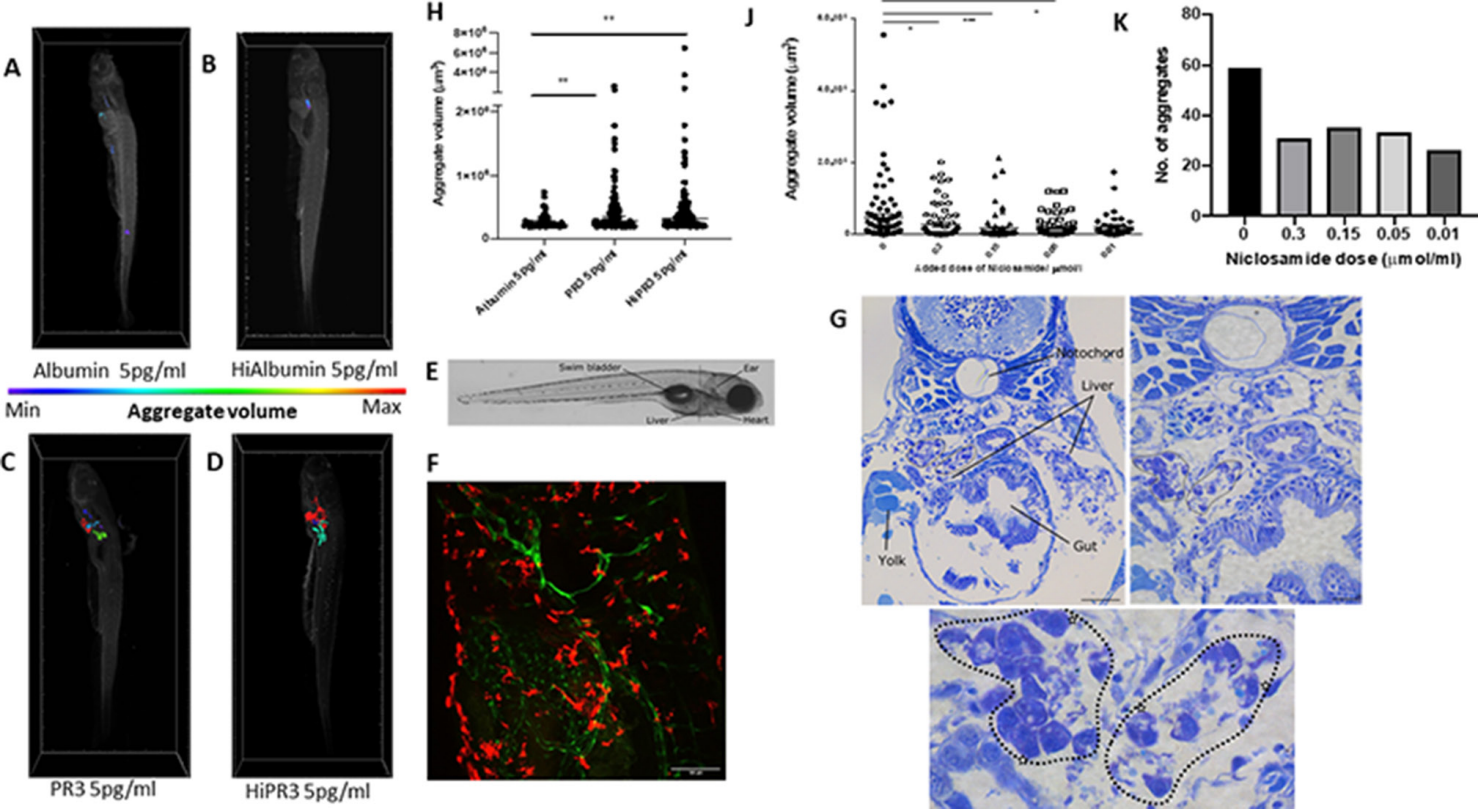
Enzymatically active and inactive PR3 stimulates macrophage aggregation and MGC formation in zebrafish and is inhibited by niclosamide. MPEg-cherry zebrafish (n=10 per group) were injected with human PR3 or albumin (intact or following heat inactivation) and imaged by lightsheet microscopy. Aggregate volumes were calculated by three-dimensional rendering of images following application of automated thresholds. Colour scale was used to demonstrate minimal and maximal aggregate volumes. Albumin with or without heat inactivation (A, B) showed minimal macrophage aggregation compared with enzymatically active and inactive PR3 at 5 pg/mL (C, D). Scale bar 100 µm. (F) Maximum projection of a 5-day-old transgenic larva (tg(*flt4*:mCitrine, *mpeg*::mCherry)) injected with PR3 into the yolk sac at 24 hours post fertilisation, at the level shown in E. The developing lymphatic system is labelled in green and peripheral macrophages are labelled in red. (E) Brightfield micrograph of a 5-day-old transgenic zebrafish larva injected with PR3 at 1 day post fertilisation, with certain anatomical structures labelled. Dashed line represents the approximate location of sections shown in G. (G) Plastic sections through the same zebrafish larva at multiple levels. MGCs can be seen (highlighted by white stars) and loose granulomas are outlined (dotted lines). Scale bars are 100 µm, 20 µm and 10 µm, respectively. Counting the number and calculating the size of the macrophage aggregates showed a significant increase in both the number and volume of macrophage aggregates in MPEg-cherry zebrafish injected with enzymatically active or inactive PR3 compared with albumin (H). Niclosamide was administered to zebrafish water following injection with PR3. Compared with PR3 alone (J, K), there was a reduction in aggregate volume and number in zebrafish injected with 0.3 µmol/mL, 0.15 µmol/mL and 0.05 µmol/mL niclosamide. Values plotted as median and 95% CI; *p<0.05, **p<0.01, ***p<0.001. Difference between conditions determined by two-way ANOVA. ANOVA, analysis of variance; Hi, heat-inactivated; MGC, multinucleated giant cells; MPeg, macrophage expressed; PR3, proteinase 3.

In order to understand granuloma formation in vivo, we developed a zebrafish embryo model. Transgenic macrophage reporter zebrafish (Tg(mpeg:GFP)) embryos at 24 hours post fertilisation (hpf) were injected into the yolk sac with human PR3 or albumin at 1 pg/mL or 5 pg/mL, either untreated or following heat inactivation. Embryos were imaged at 120 hpf with lightsheet microscopy and showed volumes of fused macrophages according to the colour scale (figure 7A–D, F and online supplemental figures 9 and 10 and supplemental video file 1). Aggregate volumes of macrophages were taken from uninjected fish to establish a normal baseline, which showed a mean of 10 248 µm^3^ and SD of 4256 µm^3^. There was low-level aggregation in albumin-injected animals, but this was significantly increased following PR3 injection at both 1 pg/mL and 5 pg/mL, with equal effect of intact or heat-inactivated PR3 (table 1 and figure 7H). Higher doses of heat-inactivated albumin or PR3 did not demonstrate greater MGC formation, but a significant difference was still seen between albumin-treated and PR3-treated fish. Imaging (figure 7F) and histological analysis of the PR3-injected fish at the level of the yolk sac, liver and gut (figure 7E) confirmed the presence of MGC and loose granulomas following staining of the fish with toluidine blue at a number of sections at low and high power (figure 7G). In subsequent experiments, injections of intact or heat-inactivated PR3 were repeated in the presence of the IL-6–signal transducer and activator of transcription 3 (STAT3) inhibitor niclosamide, added to the fish water, at different concentrations (0.01–0.3 µmol/L) (figure 7J, K), which demonstrated significant inhibition of macrophage aggregation (table 1).

## Discussion

Granulomas are organised aggregates of inflammatory cells that serve to isolate and contain certain infectious antigens or chronic irritants that are not readily eliminated through conventional immune responses. Although commonly associated with infectious diseases, a significant number of autoimmune, autoinflammatory or immunodeficient conditions result in granuloma formation in various body sites and may also serve as a final protective mechanism for the host, in many cases to unknown antigens. These may arise in some conditions, following a failure to clear more conventional infectious agents, since infectious triggers are known to precipitate many autoimmune or autoinflammatory diseases, but the reason for their persistence is not known, unless they also respond to self-antigens. At the heart of the granulomas are specialised macrophages that fuse and form into MGC, with augmented phagocytic and bactericidal capabilities. The pathogenesis of granulomas in the context of many autoimmune diseases, and particularly in ANCA-associated vasculitides, remains poorly understood, but is critically important, as persistent granulomas contribute to tissue damage and significant morbidity, resulting in exposure of patients to prolonged immunosuppressive medications, which leads to further adverse effects.^9 14 39^

While certain clinical features, such as ENT disease, are assumed to be surrogates for GPA, patients with MPA may also demonstrate sinonasal disease, but true granulomas are restricted to patients with GPA, who generally express PR3-ANCA, but variably express MPO-ANCA, especially in certain ethnic groups.

We tested the hypothesis that persistent PR3, found in greater abundance in patients with GPA and known to frustrate macrophage-mediated clearance, may be critical in promoting granuloma formation. We have shown that in vitro PR3 and not MPO promotes the greatest monocyte cell fusion and MGC formation, which is associated with elevated levels of secreted IL-6 and MCP-1. We did not find elevated levels of some of the cytokines associated with granuloma formation in the context of mycobacterial infections. Recent work from clinical cohorts has also shown significant differences in circulating IL-6 between patients with GPA and patients with MPA, supporting our findings that IL-6 is a key cytokine in mediating MGC and granuloma formation in patients with GPA.^40^ We confirmed a role of PR3 in mediating MGC even in patients with MPO-ANCA GPA, suggesting that certain cellular characteristics predispose to MGC formation in GPA, the subject of ongoing work.

That there was also an increase in MGC formation following stimulation with HNE is perhaps not so surprising as HNE shares 56% homology with PR3, has similar overall structure and shares the same three amino acid catalytic site.^41^ It differs in its electrochemical charge and along with amino acid differences results in different target specificity. PR3, unlike HNE, is closely associated with the neutrophil cell membrane and interacts with lipid bilayers in very different ways. These similarities and differences in binding surface molecules between the two may provide some clues as to other critical pathways involved in MGC formation in AAV and other immune-mediated diseases, and warrant further investigation.

Zebrafish larvae, which contain early competent macrophages differentiating in the yolk sac, have been used as established models of granuloma formation, especially in the context of mycobacterial infection.^42^ Although they lack T cells early on, they can still form macrophage aggregates, typical of granulomas. Our novel model recapitulated these macrophage aggregates and MGC formation after PR3 injection into the yolk sac, with either enzymatically active or inactive PR3. Administration of niclosamide significantly attenuated this macrophage aggregation, demonstrating that the model can serve as a useful screening tool for novel PR3-mediated granuloma therapies. However, niclosamide has effects on multiple pathways, including Wnt, oxidative phosphorylation, Notch and NFκB, and is therefore not uniquely an inhibitor of the IL-6–STAT3 pathway.^43^ Further experiments are needed to confirm the IL-6 dependence of granuloma formation in this in vivo model.

In conclusion, we demonstrate a critical role for PR3 in mediating the formation of MGC and granulomas both in vitro and in vivo, in susceptible patients with GPA, which may be in part related to greater monocyte PR3 receptor expression, allowing augmented activation.

## Data Availability

Data are available upon reasonable request.

## References

[1] Anderson JM . Multinucleated giant cells. Curr Opin Hematol 2000;7:40–7. 10.1097/00062752-200001000-00008 10608503

[2] Bosch X , Guilabert A , Font J . Antineutrophil cytoplasmic antibodies. Lancet 2006;368:404–18. 10.1016/S0140-6736(06)69114-9 16876669

[3] Gonzalez-Gay MA , Garcia-Porrua C , Guerrero J , et al . The epidemiology of the primary systemic vasculitides in northwest Spain: implications of the chapel Hill consensus conference definitions. Arthritis Rheum 2003;49:388–93. 10.1002/art.11115 12794795

[4] Ntatsaki E , Watts RA , Scott DGI . Epidemiology of ANCA-associated vasculitis. Rheum Dis Clin North Am 2010;36:447–61. 10.1016/j.rdc.2010.04.002 20688243

[5] Jennette JC , Falk RJ , Bacon PA , et al . 2012 revised international chapel hill consensus conference nomenclature of vasculitides. Arthritis Rheum 2013;65:1–11. 10.1002/art.37715 23045170

[6] Specks U , Merkel PA , Seo P , et al . Efficacy of remission-induction regimens for ANCA-associated vasculitis. N Engl J Med 2013;369:417–27. 10.1056/NEJMoa1213277 23902481 PMC5953195

[7] Walsh M , Flossmann O , Berden A , et al . Risk factors for relapse of antineutrophil cytoplasmic antibody-associated vasculitis. Arthritis Rheum 2012;64:542–8. 10.1002/art.33361 21953279

[8] Schilder AM . Wegener’s granulomatosis vasculitis and granuloma. Autoimmun Rev 2010;9:483–7. 10.1016/j.autrev.2010.02.006 20156603

[9] Henderson SR , Copley SJ , Pusey CD , et al . Prolonged B cell depletion with rituximab is effective in treating refractory pulmonary granulomatous inflammation in granulomatosis with polyangiitis (gpa). Medicine (Baltimore) 2014;93:e229. 10.1097/MD.0000000000000229 25501085 PMC4602771

[10] Lyons PA , Rayner TF , Trivedi S , et al . Genetically distinct subsets within ANCA-associated vasculitis. N Engl J Med 2012;367:214–23. 10.1056/NEJMoa1108735 22808956 PMC3773907

[11] Pagán AJ , Ramakrishnan L . The formation and function of granulomas. Annu Rev Immunol 2018;36:639–65. 10.1146/annurev-immunol-032712-100022 29400999

[12] Coury F , Annels N , Rivollier A , et al . Langerhans cell histiocytosis reveals a new IL-17A-dependent pathway of dendritic cell fusion. Nat Med 2008;14:81–7. 10.1038/nm1694 18157139

[13] Helming L , Gordon S . The molecular basis of macrophage fusion. Immunobiology 2007;212:785–93. 10.1016/j.imbio.2007.09.012 18086379

[14] Müller A , Krause B , Kerstein-Stähle A , et al . Granulomatous inflammation in ANCA-associated vasculitis. Int J Mol Sci 2021;22:6474. 10.3390/ijms22126474 34204207 PMC8234846

[15] Huugen D , Tervaert JWC , Heeringa P . Antineutrophil cytoplasmic autoantibodies and pathophysiology: new insights from animal models. Curr Opin Rheumatol 2004;16:4–8. 10.1097/00002281-200401000-00003 14673382

[16] Xiao H , Heeringa P , Hu P , et al . Antineutrophil cytoplasmic autoantibodies specific for myeloperoxidase cause glomerulonephritis and vasculitis in mice. J Clin Invest 2002;110:955–63. 10.1172/JCI15918 12370273 PMC151154

[17] Little MA , Smyth CL , Yadav R , et al . Antineutrophil cytoplasm antibodies directed against myeloperoxidase augment leukocyte-microvascular interactions in vivo. Blood 2005;106:2050–8. 10.1182/blood-2005-03-0921 15933057

[18] Schreiber A , Rolle S , Peripelittchenko L , et al . Phosphoinositol 3-kinase-gamma mediates antineutrophil cytoplasmic autoantibody-induced glomerulonephritis. Kidney Int 2010;77:118–28. 10.1038/ki.2009.420 19907415

[19] Primo VC , Marusic S , Franklin CC , et al . Anti-PR3 immune responses induce segmental and necrotizing glomerulonephritis. Clin Exp Immunol 2010;159:327–37. 10.1111/j.1365-2249.2009.04072.x 20015271 PMC2819498

[20] van der Geld YM , Hellmark T , Selga D , et al . Rats and mice immunised with chimeric human/mouse proteinase 3 produce autoantibodies to mouse PR3 and rat granulocytes. Ann Rheum Dis 2007;66:1679–82. 10.1136/ard.2006.064626 17644551 PMC2095322

[21] Wiesner O , Litwiller RD , Hummel AM , et al . Differences between human proteinase 3 and neutrophil elastase and their murine homologues are relevant for murine model experiments. FEBS Lett 2005;579:5305–12. 10.1016/j.febslet.2005.08.056 16182289

[22] Martin KR , Witko-Sarsat V . Proteinase 3: the odd one out that became an autoantigen. J Leukoc Biol 2017;102:689–98. 10.1189/jlb.3MR0217-069R 28546501

[23] Halbwachs-Mecarelli L , Bessou G , Lesavre P , et al . Bimodal distribution of proteinase 3 (PR3) surface expression reflects a constitutive heterogeneity in the polymorphonuclear neutrophil pool. FEBS Lett 1995;374:29–33. 10.1016/0014-5793(95)01073-n 7589506

[24] Witko-Sarsat V , Lesavre P , Lopez S , et al . A large subset of neutrophils expressing membrane proteinase 3 is a risk factor for vasculitis and rheumatoid arthritis. J Am Soc Nephrol 1999;10:1224–33. 10.1681/ASN.V1061224 10361860

[25] Schreiber A , Busjahn A , Luft FC , et al . Membrane expression of proteinase 3 is genetically determined. J Am Soc Nephrol 2003;14:68–75. 10.1097/01.asn.0000040751.83734.d1 12506139

[26] Wiesner O , Russell KA , Lee AS , et al . Antineutrophil cytoplasmic antibodies reacting with human neutrophil elastase as a diagnostic marker for cocaine-induced midline destructive lesions but not autoimmune vasculitis. Arthritis Rheum 2004;50:2954–65. 10.1002/art.20479 15457464

[27] Millet A , Martin KR , Bonnefoy F , et al . Proteinase 3 on apoptotic cells disrupts immune silencing in autoimmune vasculitis. J Clin Invest 2015;125:4107–21. 10.1172/JCI78182 26436651 PMC4639994

[28] Kantari C , Pederzoli-Ribeil M , Amir-Moazami O , et al . Proteinase 3, the Wegener autoantigen, is externalized during neutrophil apoptosis: evidence for a functional association with phospholipid scramblase 1 and interference with macrophage phagocytosis. Blood 2007;110:4086–95. 10.1182/blood-2007-03-080457 17712045

[29] Johansson U , Lawson C , Dabare M , et al . Human peripheral blood monocytes express protease receptor-2 and respond to receptor activation by production of IL-6, IL-8, and IL-1 { beta }. J Leukoc Biol 2005;78:967–75. 10.1189/jlb.0704422 16000389

[30] David A , Kacher Y , Specks U , et al . Interaction of proteinase 3 with CD11b/CD18 (beta2 integrin) on the cell membrane of human neutrophils. J Leukoc Biol 2003;74:551–7. 10.1189/jlb.1202624 12960243

[31] Gabillet J , Millet A , Pederzoli-Ribeil M , et al . Proteinase 3, the autoantigen in granulomatosis with polyangiitis, associates with calreticulin on apoptotic neutrophils, impairs macrophage phagocytosis, and promotes inflammation. J Immunol 2012;189:2574–83. 10.4049/jimmunol.1200600 22844112

[32] Mukhtyar C , Lee R , Brown D , et al . Modification and validation of the Birmingham vasculitis activity score (version 3). Ann Rheum Dis 2009;68:1827–32. 10.1136/ard.2008.101279 19054820

[33] Exley AR , Bacon PA , Luqmani RA , et al . Development and initial validation of the vasculitis damage index for the standardized clinical assessment of damage in the systemic vasculitides. Arthritis Rheum 1997;40:371–80. 10.1002/art.1780400222 9041949

[34] Gasser A , Möst J . Generation of multinucleated giant cells in vitro by culture of human monocytes with Mycobacterium bovis BCG in combination with cytokine-containing supernatants. Infect Immun 1999;67:395–402. 10.1128/IAI.67.1.395-402.1999 9864241 PMC96322

[35] Möst J , Spötl L , Mayr G , et al . Formation of multinucleated giant cells in vitro is dependent on the stage of monocyte to macrophage maturation. Blood 1997;89:662–71. 10.1182/blood.V89.2.662 9002970

[36] Chang D-Y , Li Z-Y , Chen M , et al . Myeloperoxidase-ANCA-positive granulomatosis with polyangiitis is a distinct subset of ANCA-associated vasculitis: a retrospective analysis of 455 patients from a single center in china. Semin Arthritis Rheum 2019;48:701–6. 10.1016/j.semarthrit.2018.05.003 29887327

[37] Ikeda S , Arita M , Misaki K , et al . Comparative investigation of respiratory tract involvement in granulomatosis with polyangiitis between PR3-ANCA positive and MPO-ANCA positive cases: a retrospective cohort study. BMC Pulm Med 2015;15:78. 10.1186/s12890-015-0068-1 26223225 PMC4520074

[38] Schirmer JH , Wright MN , Herrmann K , et al . Myeloperoxidase-antineutrophil cytoplasmic antibody (ANCA) -positive granulomatosis with polyangiitis (Wegene’'s) is a clinically distinct subset of ANCA-associated vasculitis: a retrospective analysis of 315 patients from a German vasculitis referral center. Arthritis Rheumatol 2016;68:2953–63. 10.1002/art.39786 27333332

[39] Hilhorst M , van Paassen P , Tervaert JWC , et al . Proteinase 3-ANCA vasculitis versus myeloperoxidase-ANCA vasculitis. J Am Soc Nephrol 2015;26:2314–27. 10.1681/ASN.2014090903 25956510 PMC4587702

[40] Berti A , Warner R , Johnson K , et al . The association of serum interleukin-6 levels with clinical outcomes in antineutrophil cytoplasmic antibody-associated vasculitis. J Autoimmun 2019;105:102302. 10.1016/j.jaut.2019.07.001 31320177 PMC7217333

[41] Hajjar E , Broemstrup T , Kantari C , et al . Structures of human proteinase 3 and neutrophil elastase -- so similar yet so different. FEBS J 2010;277:2238–54. 10.1111/j.1742-4658.2010.07659.x 20423453

[42] Davis JM , Clay H , Lewis JL , et al . Real-Time visualization of mycobacterium-macrophage interactions leading to initiation of granuloma formation in zebrafish embryos. Immunity 2002;17:693–702. 10.1016/s1074-7613(02)00475-2 12479816

[43] Chen W , Mook RA , Premont RT , et al . Niclosamide: beyond an antihelminthic drug. Cell Signal 2018;41:89–96. 10.1016/j.cellsig.2017.04.001 28389414 PMC5628105

